# Comparative Metagenomic Analysis of the Gut Microbiota of Captive Pangolins: A Case Study of Two Species

**DOI:** 10.3390/ani15010057

**Published:** 2024-12-30

**Authors:** Zhengyu Dai, Bowen Xie, Chungang Xie, Jinsuo Xiang, Xinmei Wang, Jing Li, Rongquan Zheng, Yanni Wang

**Affiliations:** 1 College of Life Sciences, Zhejiang Normal University, Jinhua 321004, China; 15381717783@163.com (Z.D.);; 2 Wildlife Protection and Management Station, Jinhua Municipal Bureau of Planning and Natural Resources, Jinhua 321052, China; 3 College of Ecology and Agriculture, Sichuan Minzu College, Chengdu 626001, China; 4 Key Lab of Wildlife Biotechnology, Conservation and Utilization of Zhejiang Province, Zhejiang Normal University, Jinhua 321004, China

**Keywords:** *Manis pentadactyla*, *Manis javanica*, gut microbiota, metagenomics, metabolic pathways

## Abstract

Pangolins, one of the most trafficked mammals, face significant health challenges in captivity, including digestive disorders and immune dysfunctions. These issues are closely linked to alterations in their gut microbiota, which play vital roles in the host metabolism, immunity, and overall health. This study investigated the differences in the gut microbiota composition and function between two pangolin species, Chinese pangolins (*Manis pentadactyla*) and Malayan pangolins (*Manis javanica*), under identical captive conditions to better understand their ecological adaptability and health implications. Using metagenomic sequencing, fecal samples from eight adult captive pangolins were analyzed, including four male Malayan pangolins and three male and one female Chinese pangolins. Comparative analyses of the alpha and beta diversities, microbial community structure, and functional profiles were performed. Both species harbored gut microbiota dominated by Firmicutes, Bacteroidetes, and Proteobacteria. However, the Chinese pangolins exhibited higher microbial diversity (Shannon index, *p* = 0.042; Simpson index, *p* = 0.037) and lower relative abundance of Proteobacteria compared with the Malayan pangolins. A functional analysis revealed significant differences in the metabolic pathways, where the Chinese pangolins demonstrated a higher potential for fiber degradation, whereas the Malayan pangolins exhibited elevated levels of antibiotic resistance genes and pathogenic taxa, such as *Escherichia coli*. These findings suggest that captivity duration and environmental stress likely contribute to the observed differences, with the Malayan pangolins experiencing greater dysbiosis due to longer captivity periods. This study provides valuable insights into the role of gut microbiota in pangolin health and offers a foundation for improving conservation strategies and captive care protocols.

## Simple Summary

Pangolins, threatened by their special diet and high extinction risk, face physiological challenges in captivity, such as digestive diseases and immune alterations. This study examined the gut microbiota of captive *Manis pentadactyla* and *Manis javanica* using metagenomic techniques to comprehend their structure, function, and influence on host health. The key findings revealed that both species predominantly harbored Firmicutes, Proteobacteria, and Bacteroidetes, where *Manis pentadactyla* exhibited a greater species diversity and lower levels of Proteobacteria. A functional analysis identified 45 metabolic pathways crucial for digesting their specialized diets, and significant differences in antibiotic resistance mechanisms were observed between the species. This study concluded that environmental and dietary factors significantly shaped the gut microbiota, which impacted the pangolins’ health. These insights provided valuable data for improving captive care, conservation strategies, understanding the ecological adaptability of pangolins, and emphasizing the necessity of customized dietary and health management protocols.

## 1. Introduction

Pangolins, as scaly anteaters, are among the most endangered mammals globally, facing a significant risk of extinction [1]. Their foraging habits in natural habitats are highly specialized, focusing predominantly on consuming chitin-coated ants and termites [2]. Listed as critically endangered on the International Union for Conservation of Nature (IUCN) Red List of Threatened Species [3], pangolins in captivity are particularly susceptible to gastrointestinal disturbances, diarrheal conditions, and parasitic infestations [4,5]. A primary factor leading to these health complications stems from the insufficient understanding of the molecular mechanisms implicated in their digestive processes, which are aggravated by the adverse impacts of artificial diets on their gastrointestinal physiology [6]. Moreover, the stress imposed by captive environments may provoke chronic stress in pangolins, potentially perturbing their immune function and altering the composition of their gut microbiota [7,8]. However, studies on the gut microbiota of pangolins remain highly limited, particularly in terms of comparative analyses between different species, such as *Manis pentadactyla* and *Manis javanica*, under identical captive conditions.

The gut microbiota, which influences host metabolism, nutrition, and immunity, plays a significant role in host health and disease [9,10,11,12]. Studies indicated that differences in lifestyle between wild and captive conditions can lead to weakened digestive and immune functions of the gut microbiota during captivity [13], suggesting that captivity can adversely affect the gut microbiota of animals. Presently, research of the gut microbiota in endangered animals in captivity has become an essential field for animal conservation [14,15]. Regarding pangolins, also endangered animals, researchers have already revealed the major microbial taxa in their gut through high-throughput sequencing technology [16], mainly including Firmicutes, Proteobacteria, and Actinobacteria. The highest mean abundance is Firmicutes, and the second is Proteobacteria [17]. These microbial communities not only participate in the digestive process of pangolins but may also affect their immune systems and behavior. Notably, certain microbial groups in the guts of pangolins, such as *Lactobacillus* and *Bacteroides*, were proven to be closely related to food digestion and host health. *Lactococcus* may be involved in the breakdown of lactose and other sugars, producing lactic acid, which helps maintain an acidic environment in the gut, inhibiting the growth of harmful bacteria. *Bacteroides* serve in the degradation of polysaccharides and the production of short-chain fatty acids, with benefits to the host’s energetic metabolism and gut health [18,19]. In addition, the studies found significant differences in the composition of gut microbiota between captive and wild Malayan pangolins, possibly due to changes in captive environments and diet. For example, in captive Malayan pangolins, the relative abundance of Proteobacteria (e.g., *Escherichia coli*) significantly increases, while Firmicutes (e.g., *Clostridium*) decreases, likely due to stress and insufficient dietary fiber in captivity. In contrast, wild Malayan pangolins exhibit a more stable gut microbiota, with higher proportions of functional genera, such as Bacteroides and Lactobacillus, which better adapt to the complex carbohydrates and fibers in their natural diet [20]. However, few studies have compared the gut microbiomes of different pangolin species under the same captivity conditions, and the molecular mechanisms of their specialized myrmecophagous habits need to be further explored.

Metagenomics refers to the microbial research method of sequencing all genomes and screening functional genes in a specific environment, serving as an important means of studying microorganisms [21]. To better protect captive pangolins and promote research on their physiology and ecology, we utilized metagenomic technology to analyze the composition, function, and adaptability of the gut microbiota in Chinese and Malayan pangolins. By analyzing the metagenomes of pangolin fecal samples, researchers can predict and contrast the functional genes and metabolic pathways of different pangolin gut microbial communities. This approach can reveal not only the species composition of microbial communities but also insights into how microorganisms adapt to the host’s physiological needs and environmental changes [22,23,24]. Metagenomic data can also help identify microbes associated with specific functions, such as those involved in degrading chitin, a substance found in insect exoskeletons. This is crucial for understanding how pangolins adapt to their specialized diets [25,26]. Moreover, the application of metagenomics can provide scientific evidence for the health management and disease prevention of captive pangolins, thus supporting pangolin treatment and conservation efforts.

To better understand the physical condition of the two species of pangolins in captivity, this study aimed to investigate the compositional and functional differences in the gut microbiota of *Manis pentadactyla* and *Manis javanica* under identical captive conditions. Specifically, we tried to compare the gut microbial diversity between the two species, identify key functional pathways and microbial taxa associated with dietary adaptation, and evaluate the potential impact of captivity on gut microbiota composition. We hypothesized that there were significant differences in the gut microbiota between the two pangolin species. Additionally, the Chinese pangolins, which had been kept in captivity for a shorter period, would exhibit higher microbial diversity and more fiber-degrading taxa due to dietary and environmental differences, while the Malayan pangolins were anticipated to display a higher abundance of pathogenic bacteria, reflecting captivity-induced stress. This research enhanced the understanding of pangolin’s ecology and provided a scientific basis for improving conservation strategies and captive management practices.

## 2. Materials and Methods

Chinese pangolins (*Manis pentadactyla*) and Malayan pangolins (*Manis javanica*), as critically endangered species, have very limited numbers in captivity, mostly relying on wild rescues. The four adult Chinese pangolins and four adult Malayan pangolins employed in this study originated from the Zhejiang Pangolin Conservation and Breeding Research Base, situated in Jinhua City. Of the eight individuals, only one Chinese pangolin was female, and all the others were male. All the Chinese pangolins were rescued from the wild in Zhejiang Province, with a rescue duration of about two years. The Malayan pangolins were intercepted from smuggling by Zhejiang Customs, with a rescue duration of about five years. All eight pangolins were individually housed in rooms that measured 6.0 m × 3.5 m, with wooden nest boxes for concealment and burrowing setups to satisfy their burrowing habits. The rooms were equipped with air conditioning and underfloor heating to maintain a constant temperature of 26.0 ± 2.0 °C and humidity at 60.0 ± 5.0%, ensuring their living and physiological needs. All pangolins received equal care during captivity, and their physical conditions were good. The pangolins were considered to be in good physical condition, as assessed by veterinary examinations. The parameters included a stable body weight, the absence of visible injuries or illnesses, normal activity levels, and a healthy appetite. These criteria are consistent with established standards for assessing the fitness of captive wildlife, as detailed in Table 1. However, due to the small sample size, this study was more inclined toward a case study, and the data obtained will be useful for future research.

Upon veterinary confirmation of the pangolins’ fitness, active circadian rhythms, and good health, we entered the pangolin enclosure every half hour from 20:00 to 05:00 to check the defecation status, and immediately carried out sterile collection if there was defecation. A disposable sterile spoon was used to collect the middle part of a fresh stool sample that had not been in contact with the air and ground. Aseptically, 1–3 g of uncontaminated internal fecal samples was transferred to 20 mL sterile sampling cups (B-CYB20, BKMAN), labeled promptly, and frozen in liquid nitrogen. These were then transported to the lab and stored in an ultra-low temperature freezer (Forma 702, Thermo Fisher Scientific, Waltham, MA, USA) at −80 °C for future analysis.

For the metagenomic sequencing, total genomic DNA was extracted from the fecal samples, and whole-genome metagenomic sequencing was performed to capture the complete microbial DNA. The genomic DNA of the fecal microbiota of the eight pangolins was extracted using the Mag-MK Soil and Stool Genome DNA Extraction Kit (B618763, Sangon Biotech, Shanghai, China). The total DNA was eluted in 50 μL of an elution buffer and stored at −80 °C prior to sequencing. All experimental protocols were strictly followed per the kit guidelines. The quality assessment of the extracted DNA was conducted through fluorescence quantitative PCR and agarose gel electrophoresis. Post-quality control, the DNA was utilized to construct sequencing libraries with the TruSeq Nano DNA LT Sample Preparation Kit (FC-121-4001, Illumina, San Diego, CA, USA). The resultant libraries were sequenced on the Illumina HiSeq 4000 platform using the PE50 strategy for paired-end sequencing [27]. The obtained data were quality controlled and then used for the bioinformatics analysis.

Raw data were preprocessed using Trimmomatic software (version 0.36) to obtain high-quality sequences [28]. The sequences in the gene sets were annotated via DIAMOND blast [29], selecting matches with the lowest e-value. MEGAN software (version 5) was used to determine the species’ relative abundances and gene abundances in each sample [30]. Using R software (version 3.2.0), the abundance data were statistically analyzed across taxonomic levels, which culminated in species composition and abundance distribution tables. Alpha diversity indices, including Good’s coverage, Shannon, Simpson, Chao1, and ACE, were calculated using the “VEGAN” package (version 2.6-2) in R software (version 3.2.0) to evaluate species diversity and richness within each specimen’s gut microbiota [31]. Beta diversity was assessed using Bray–Curtis distance algorithms in the “betapart” package of R software (version 3.2.0) to obtain distance matrices for PCoA (Principal Coordinates Analysis) and NMDS (Non-metric Multidimensional Scaling Analysis) [32]. The PCoA and NMDS could intuitively visualize and analyze the microbial community dissimilarities among the pangolins. A PCoA, a linear dimensionality reduction technique, projects high-dimensional data onto a lower-dimensional space, effectively reflecting the relative distances between samples. An NMDS, on the other hand, is a non-linear dimensionality reduction method that iteratively optimizes the placement of samples in a low-dimensional space to minimize the discrepancy between the distances in this reduced space and the original high-dimensional space, thus revealing the non-linear relationships between samples. In essence, a closer proximity between samples signifies a greater similarity in their gut microbiota composition, while larger distances suggest more substantial differences in community species abundance. A PERMANOVA (non-parametric multivariate analysis of variance) and Anosim (analysis of similarities) were used to evaluate the significance of similarity and differences in gut microbial community structure between the samples and groups.

## 3. Results

### 3.1. Metagenomic Data

Following the elimination of substandard and contaminant hosts, we obtained 102.79 GB of high-quality data, averaging 12.84 GB per sample. A non-redundant database of 2,617,078 open reading frames (ORFs) was created with an average of 327,135 per sample, indicating an effective assembly.

### 3.2. Gut Microbiota Composition and Structure of Two Pangolin Species

#### 3.2.1. Gut Microbiota Composition

The dominant bacterial phyla in the gut microbiota of the four Chinese pangolins were *Firmicutes* (61.99%), *Proteobacteria* (13.08%), *Bacteroidetes* (13.03%), and *Actinobacteria* (0.21%). In addition, Uroviricota, a phylum of tailed bacteriophages, contributed 3.81%. For the four Malayan pangolins, Proteobacteria (53.01%) was predominant, followed by *Firmicutes* (33.99%), *Bacteroidetes* (5.99%), and *Actinobacteria* (0.13%), with *Uroviricota* comprising 3.76%. Significant inter-individual variations were observed in both groups.

Significant variations were noted at the genus level. *Clostridium* (27.58%) was predominant in the Chinese pangolins, followed by *Bacteroides* (10.14%), *Escherichia* (9.13%), *Roseburia* (1.96%), *Lactococcus* (1.94%), and *Proteus* (1.87%). Notably, Clostridium represented approximately half (55 ± 4%) of the gut microbiota in three male Chinese pangolins. In the Malayan pangolins, *Escherichia* dominated with 45.19% of the total, followed by *Clostridium* (15.86%), *Bacteroides* (5.74%), *Lactococcus* (4.82%), *Shigella* (3.43%), *Roseburia* (1.47%), *Seuratvirus* (1.93%), *Proteus* (1.18%), and *Salmonella* (1.01%).

#### 3.2.2. Alpha Diversity

**Figure 1:**
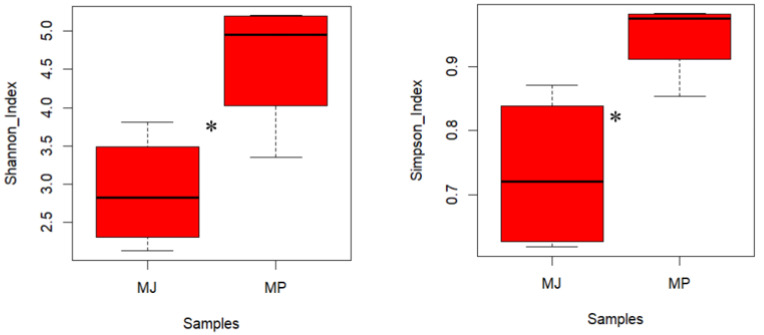
Comparison between groups of Shannon and Simpson diversity indices across two species of pangolin utilizing boxplot plots. The figures show the distribution of diversity indices in each group and whether there were significant differences in the diversity indices between the groups. The horizontal axis represents the different groups (MJ—*Manis pentadactyla*; MP—*Manis javanica*). The vertical axis represents the index values. “*” represents a statistically significant difference between the two species of pangolins.

Alpha diversity indices effectively indicate the extent of species diversity within the samples. The Chao1 index (Chao1 richness estimator) and ACE index (Abundance-based Coverage Estimator) estimate the actual number of species in a community and are often used to represent richness [33]. The Shannon diversity index takes into account both the species richness and evenness [34], while the Simpson diversity index is sensitive to evenness and the dominance of species within the community [35]. Higher values of these indices indicate a greater diversity and richness of the microbiota. The specific results are shown in Table 2. The alpha diversity results show that the Chao1 and ACE indices, which represented the community richness index between two pangolin species showed no significant difference (*p* = 0.059 and *p* = 0.067, respectively). The Shannon and Simpson diversity indices of the gut microbiota in the Chinese pangolins were significantly higher than those in the Malayan pangolins (*p* = 0.042 and *p* = 0.037, respectively), indicating that the gut microbiota of the Chinese pangolins had higher species diversity, evenness, and actual species number. The boxplot in Figure 1 further illustrates the distribution of these diversity indices, highlighting significant differences between the groups. Overall, the levels of the four indices for the Chinese pangolin were slightly higher than those for the Malayan pangolin.

#### 3.2.3. Beta Diversity

To evaluate the extent of the microbial community variation between the different groups, the beta diversity, a crucial metric for assessing the differences in structure and composition in gut microbiota, was employed.

**Figure A1:**
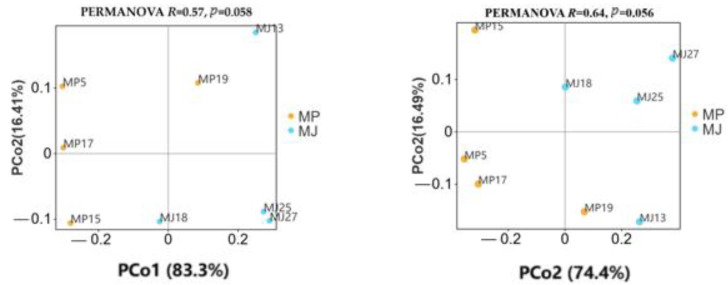
The PCoA (95% confidence interval) and PERMANOVA results at different classification levels between the MP and MJ groups.

**Figure A2:**
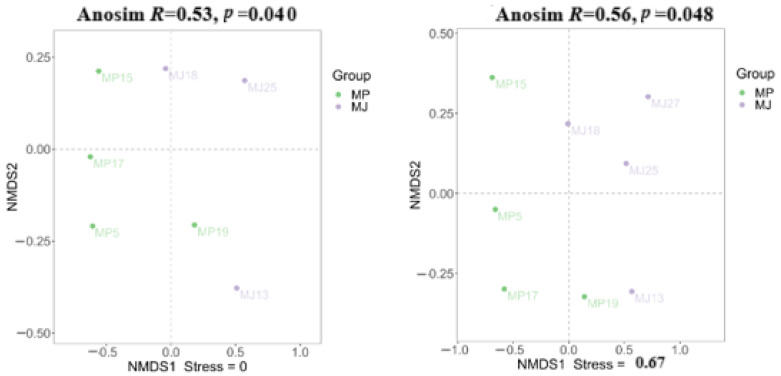
The NMDS (95% confidence interval) and Anosim results at different classification levels between the MP and MJ groups.

**Figure A3:**
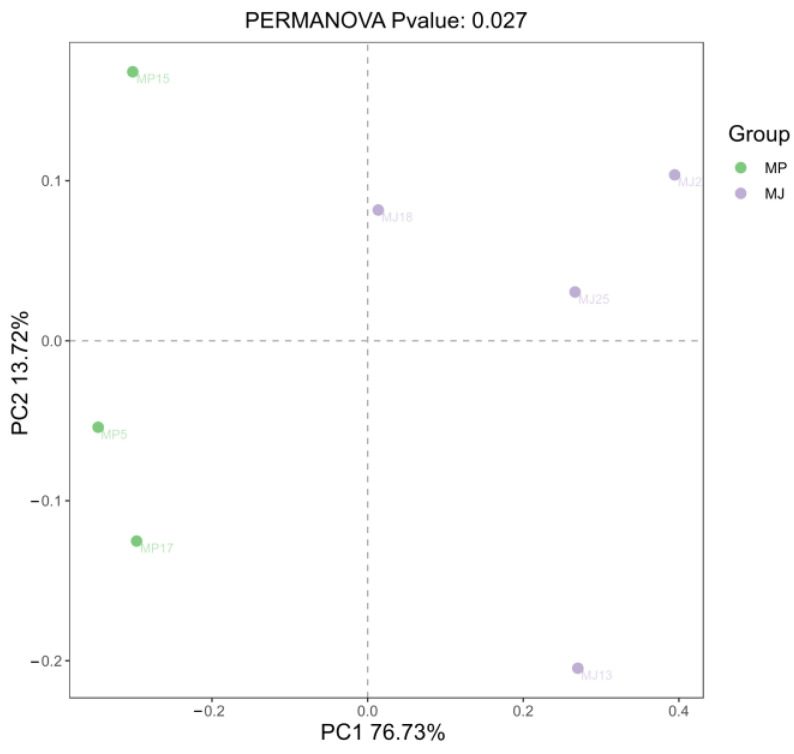
The PCoA (95% confidence interval) and PERMANOVA results at the genus level between the MP and MJ groups (without MP19).

In the context of ecological statistics, a PCoA and NMDS were employed to statistically assess whether the microbial community structures exhibited significant variations across the different groups. Our findings reveal that the PCoA plots for the two pangolin species did not display any evident separation at the phylum and genus levels (Figure A1). Additionally, PERMANOVA tests for both the phylum- and genus-level microbiota communities yielded *p*-values that exceeded 0.05 (*p* = 0.058 and *p* = 0.056, respectively). These results collectively suggest that the structural composition of the gut microbiota in the two pangolin species did not exhibit significant differences. Intriguingly, the NMDSs at both the phylum and genus levels unveiled a distinct pattern of separation between the two pangolin species (Figure A2), indicating a significant dissimilarity in their gut microbial communities (*p* = 0.040 and *p* = 0.048, respectively). Moreover, the inter-group variation was more pronounced than the intra-group variation. After careful examination, we observed a consistent pattern within the Chinese pangolin samples, that is, MP5, MP15, and MP17 clustered closely, while MP19 was positioned further away. Interestingly, when the female sample MP19 was excluded, the gut microbial community composition of the two pangolin species exhibited more divergence (Figure A3). This result suggests that gender might also be a contributing factor influencing intra-species variations in gut microbiota composition.

Therefore, combining the results of the above four statistical analyses indicated that there were certain differences in the structure of the gut microbiota communities between the Chinese pangolin and Malayan pangolin groups, but the differences showed no significance (phylum: R = 0.32, *p* = 0.058; genus: R = 0.41, *p* = 0.056; based on PERMANOVA analysis), and the source of these differences was inter-group rather than intra-group (phylum: R = 0.53, *p* = 0.040; genus: R = 0.56, *p* = 0.048; based on Anosim).

### 3.3. Analysis of Differences in Gut Microbiota Structure Between Two Pangolin Species

**Figure 2:**
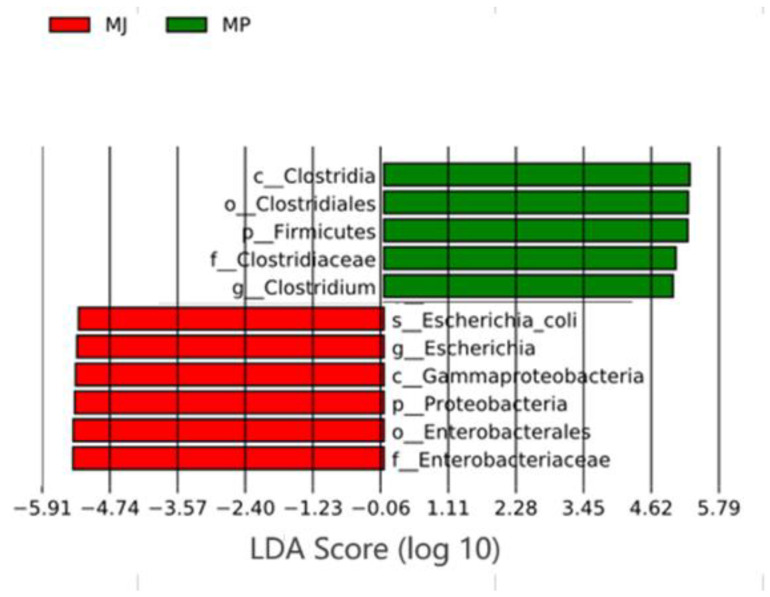
Differential of gut microbiota species compositions in the two pangolin species by LEfSe analysis. The figure shows the scores of the differential species, with different colors representing different groups. The red bars indicate the species with a relatively higher abundance in the Malayan pangolins, while the green bars indicate the species with a relatively higher abundance in the Chinese pangolins.

LEfSe (Linear discriminant analysis coupled with Effect Size) analysis was used to reveal statistically significant differential species in the gut microbiota of the two pangolin species. Based on the relative abundance spectrum of the species, a significant difference analysis of the microbial communities at all taxonomic levels of the Chinese pangolin and the Malayan pangolin was conducted using the Wilcoxon rank-sum test, and biomarkers with an LDA score ≥ 4 and *p* < 0.05 were selected to plot a bar chart. As shown in Figure 2, the LDA scores of 11 biomarkers between the Chinese pangolins and Malayan pangolins were greater than 4. According to the LDA scores, the abundances of c_Clostridia, o_Clostridiales, p_Firmicutes, f_Clostridiaceae, and g_Clostridium in the Chinese pangolin were significantly higher than in the Malayan pangolin group (*p* < 0.05, LDA ≥ 4), while the abundances of f_Enterobacteriaceae, o_Enterobacterales, p_Proteobacteria, c_Gammaproteobacteria, g_*Escherichia*, and s_*Escherichia_coli* in the Malayan pangolin group were significantly higher than in Chinese pangolin group. It should be noted that most of the microorganisms with high abundances in the Malayan pangolin were pathogenic.

### 3.4. Functional Differences in Gut Microbiota Between Two Pangolin Species

#### 3.4.1. Functional Groups and Metabolic Pathways Statistics and Composition Analysis

**Figure 3:**
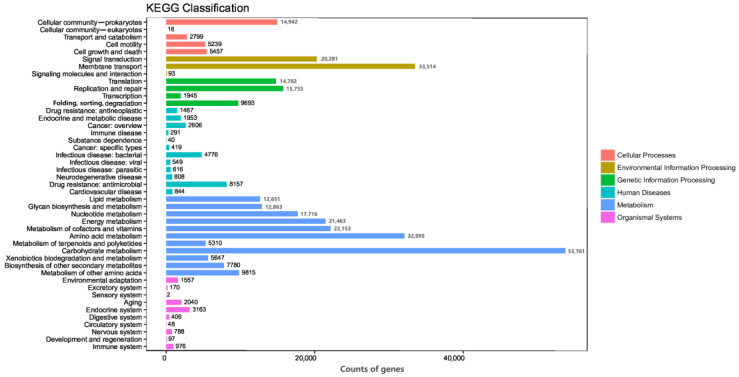
KEGG database gene annotation count chart. The numbers on the bars represent the non-redundant gene numbers annotated. Different colors represent the KEGG level 1 functional categories, with annotations detailed in the corresponding legend.

The KEGG database was used to annotate the pathways of different levels and abundances. The results show that a total of 418,224 genes were annotated in the non-redundant gene set, covering 371 pathways across six major categories, and ranked by the relative abundances of the enriched genes from the highest to the lowest as follows: metabolism (48.12%), environmental information processing (12.88%), genetic information processing (10.08%), cellular processes (6.80%), human diseases (5.39%), and organismal systems (2.25%). The gut microbiota of the two pangolin species played an important role in carbohydrate metabolism (12.85%), membrane transport (8.01%), amino acid metabolism (7.67%), cofactor and vitamin metabolism (5.3%), and energy metabolism (5.13%). In environmental information processing, membrane transport and signal transduction were the main functions. Additionally, in genetic information transfer, replication and repair; translation; and folding, sorting, and degradation were key processes. Prokaryotic cell immunity was an important aspect of cellular processes, as detailed in Figure 3.

**Figure 4:**
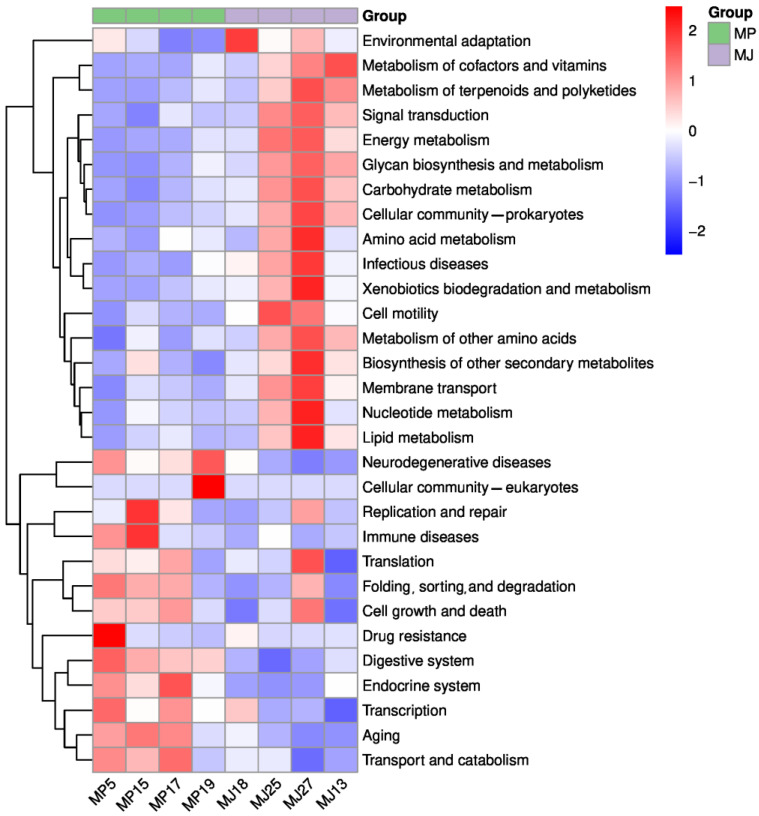
Heat map of the gut microbial functional composition in the eight pangolin individuals. The horizontal axis represents the sample information; the vertical axis represents the functional annotation information. The clustering tree on the left represents the functional clustering; the clustering branches above represent the samples from the different groups, with green representing the Chinese pangolins and purple representing the Malayan pangolins. Orange indicates a higher functional relative abundance, and blue indicates a lower functional relative abundance.

**Figure A4:**
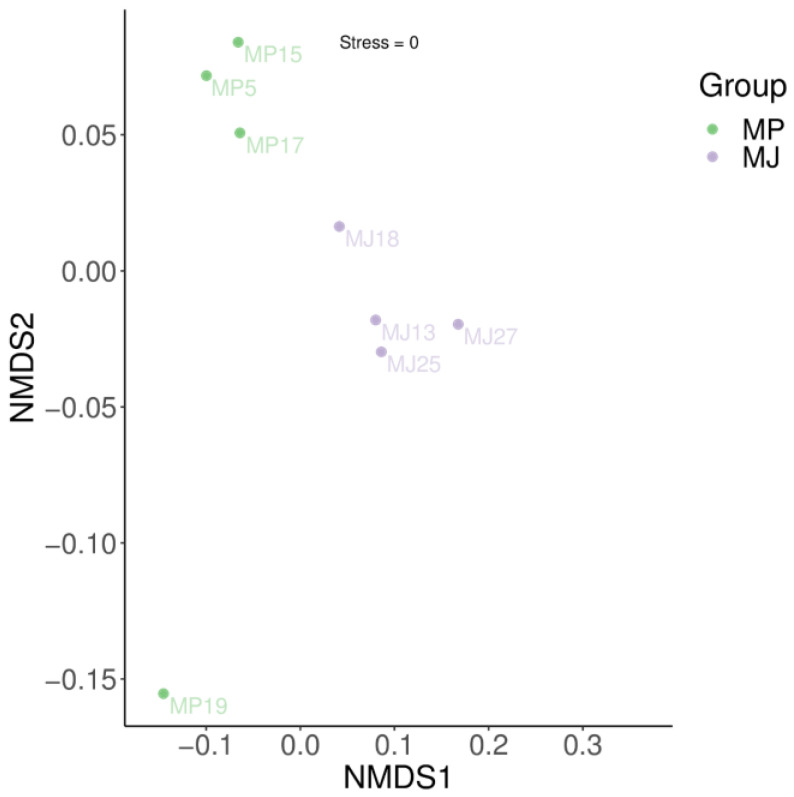
NMDS of KEGG level 2 in two groups of pangolins. Each point represents a sample, with the distance between points indicating the degree of difference between samples. Samples from the same group are represented by the same color; stress is the stress coefficient, indicating the accuracy of the analysis. When the stress is less than 0.2, an NMDS is considered reliable.

Analysis of the relative abundance distribution of the functional pathways revealed some differences between the two pangolin species (Figure 4). From the functional abundance heat map in Figure 4, the relative abundances of the Malayan pangolins in cell processes and human diseases were higher, while the relative abundances of the Chinese pangolins exhibited more prominently in organ systems and the processing of genetic information. These results suggest potential functional divergence in the gut microbiota composition of the two species. Further supporting this notion, the NMDS at KEGG level 2 revealed significant intergroup differences (Figure A4) with a perfect stress value of 0, indicating ideal data representation. The Anosim at KEGG level 2 confirmed the conclusion, demonstrating that intergroup differences were statistically more significant than intragroup variations (R = 0.562, *p* = 0.035).

#### 3.4.2. CAZy Functional Annotation

**Figure A5:**
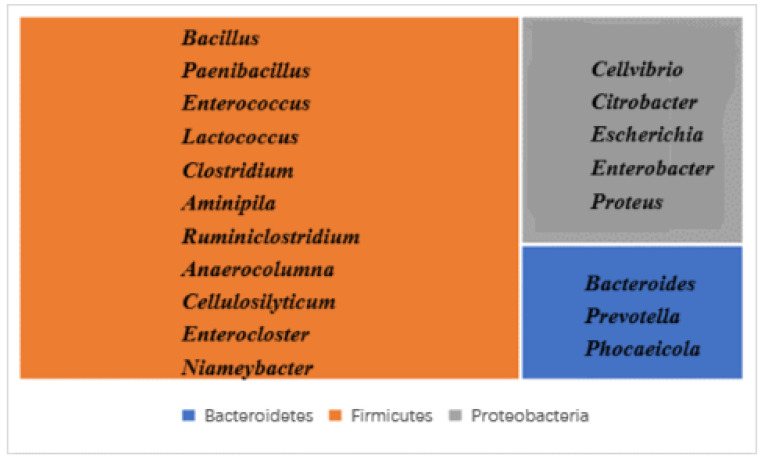
Bacteria involved in chitin degradation in the guts of pangolins.

How pangolins digest the chitin shells of ants has been an interesting topic. As a polysaccharide, the degradation of chitin requires carbohydrate-active enzymes (CAZymes). Therefore, we compared sequenced gene sets within the CAZy database, annotating 22,149 genes into six functional categories (Figure A5). Notably, the glycoside hydrolases (GHs) family harbored the most genes, where it encompassed key enzymes for chitin degradation, namely, chitinase, chitosanase, N-acetylglucosaminidase, and chitin deacetylase. The high abundance of these enzymes was associated with the pangolins’ specialized food habits and highlighted the crucial role of GHs in their myrmecophagous behavior.

**Figure 5:**
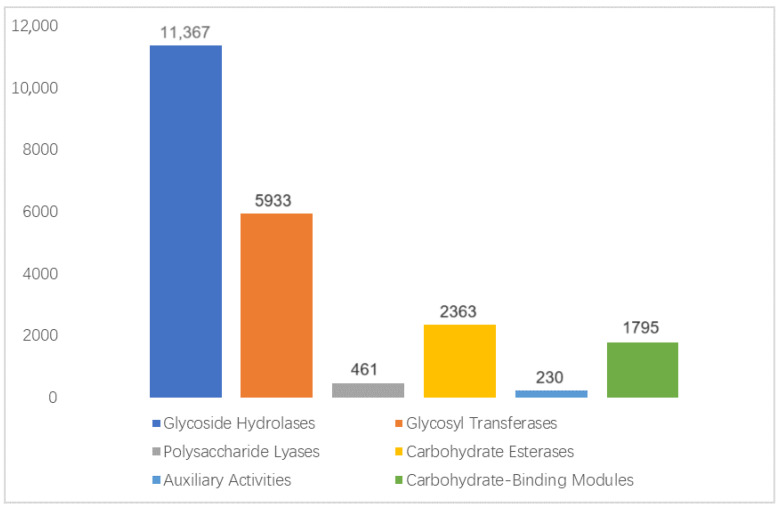
The statistical chart of the gene numbers annotated by the CAZy database. The numbers on the bars represent the non-redundant gene numbers annotated; the coordinate axis marks the CAZy class functional categories, mainly covering six major functions: glycoside hydrolases (GHs), glycosyl transferases (GTs), polysaccharide lyases (PLs), carbohydrate esterases (CEs), auxiliary activities (AAs), and carbohydrate-binding modules (CBMs).

Further analysis of the non-redundant gene set annotations revealed the presence of bacterial genera associated with chitin degradation within Firmicutes, Bacteroidetes, and Proteobacteria (Figure 5). These bacterial genera were consistent with the most abundant bacterial communities observed in both the pangolin species. However, considering the previously established differences in the gut microbiota compositions between the two pangolin species, we hypothesized that while both species had chitinolytic capabilities, their chitin preferences might differ, potentially influencing the dietary composition.

#### 3.4.3. CARD Resistance Analysis

**Figure 6:**
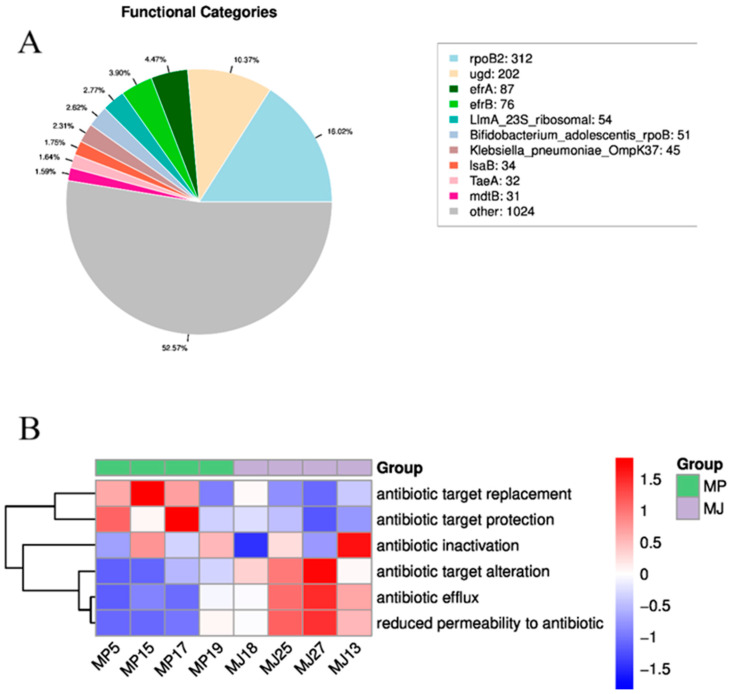
(**A**) Top 10 ARO annotations from the CARD database. Different colors in the pie chart represent the proportion of genes annotated to the respective ARO, with the number of genes annotated to the ARO in the legend at the left top. (**B**) The functional abundance heat map of resistance mechanisms in the two pangolin species. The heat map indicates the relative abundance of bacterial strains or genes with specific resistance mechanisms within the microbial community. Orange indicates a higher functional relative abundance, and blue indicates a lower functional relative abundance.

The CARD resistance gene database, organized around the ARO (Antibiotic Resistance Ontology), provides annotations for resistance gene functions. As shown in Figure 6A, we annotated 1949 resistance genes in the eight pangolin samples, which belonged to 1048 ARGs. The most annotated ARGs were rpoB2 and ugd, which are resistant to rifampin and polymyxin, respectively. The functional composition heat map in Figure 6B shows that in terms of resistance mechanisms, the Malayan pangolins had higher relative abundances of antibiotic target alteration, antibiotic extrusion, and decreased antibiotic permeability mechanisms, while the Chinese pangolins had higher relative abundances of antibiotic target replacement and antibiotic target protection. The Anoism analysis also confirmed this conclusion (R = 0.656, *p* = 0.03), indicating significant differences in the antibiotic resistance strategies between the gut microbiota of the two pangolin species. Understanding these mechanisms is crucial for developing effective treatment protocols for pangolins in the future.

## 4. Discussion

With the continuous advancement of molecular biology techniques, it has become increasingly recognized that the gut microbiota plays a crucial role in animal health. This study employed metagenomics to compare the gut microbiota of captive Chinese pangolins and Malayan pangolins, with the aim to investigate their composition, functionality, and potential associations with host digestion, immune response, and behavior. The objective was to provide valuable insights for pangolin conservation and health management strategies. The findings reveal significant differences in the composition and functional traits of the gut microbiota between the two species, underscoring the distinct microbial landscapes in these animals. Consistent with previous research [36,37], Firmicutes, Bacteroidetes, and Proteobacteria were identified as the dominant phyla in both species. Firmicutes are primarily involved in the digestion of cellulose and other complex carbohydrates, while Bacteroidetes are generally associated with lipid metabolism and gut barrier function. Notably, Malayan pangolins exhibited a significantly higher abundance of Proteobacteria compared with prior studies on wild pangolins [38]. Proteobacteria include beneficial bacteria involved in nitrogen and sulfur cycling and organic matter degradation, but they also harbor potential pathogens [39], indicating a multifaceted role in pangolin gut metabolism and serving as a marker for ecological imbalance [40]. The elevated relative abundance of Proteobacteria in the Malayan pangolins suggested potential dysbiosis, likely driven by the long-term reliance on artificial feed, which may have altered the gut microbiota to adapt to new dietary demands. At the genus level, pathogenic genera, such as *Escherichia*, *Shigella*, and *Salmonella*, were also detected in the Malayan pangolins. These findings suggest that long-term captivity and associated stress might lead to an imbalance in the gut microbiota, promoting the proliferation of opportunistic pathogens. In contrast, the gut microbiota of Chinese pangolins showed relatively lower abundances of these pathogenic genera, likely due to their shorter captivity duration and reduced cumulative stress exposure. This highlighted the potential role of captivity duration as a critical factor influencing the gut microbiota composition and the emergence of pathogenic bacteria.

Differences in the gut microbiota composition also had a profound impact on immune function and overall health in the two species. The diversity analysis indicated that the Chinese pangolins harbored higher species richness, evenness, and overall bacterial diversity. In contrast, the gut microbiota of the long-term captive Malayan pangolins was more stable but exhibited reduced diversity, with a notably higher abundance of potential pathogens, such as *Escherichia coli*. This imbalance might reflect weakened immune function due to prolonged captivity [41]. Various factors, including the duration of captivity, dietary modifications [42], and environmental conditions [43], were likely to have collectively contributed to these microbial differences. Previously, Yan et al. conducted 16S rRNA sequencing and cortisol measurements on fecal samples of Malayan pangolins rescued from confiscation and kept in captivity for 2–4 years [7]. Their findings revealed that chronic stress significantly affected both the diversity of the fecal microbiota and cortisol levels in Malayan pangolins. A comparative analysis of the fecal microbiota diversity between short-term captive and confiscated Malayan pangolins showed that the Shannon index and observed OTUs of the long-term rescued individuals were significantly lower than those of the short-term captive group [20]. Based on these findings, we hypothesize that in this study, under identical artificial captive conditions, the longer captivity duration of the Malayan pangolins (over five years) likely resulted in higher stress levels compared with the Chinese pangolins (kept in captivity for 2–2.5 years). The elevated stress might have contributed to the reduced diversity of the gut microbiota in Malayan pangolins.

In addition to immune function, these compositional differences in the gut microbiota may significantly influence nutritional metabolism, impacting growth, development, and reproductive capacity. For instance, *Clostridium* was the most abundant genus in the Chinese pangolins and is known to play a crucial role in several metabolic pathways, including cellulose degradation [44]. In contrast, the Malayan pangolins exhibited a higher abundance of *Escherichia*, which is involved in nitrogen cycling and amino acid metabolism. Among the shared bacterial genera, *Bacteroides* is associated with carbohydrate metabolism, particularly the degradation of indigestible plant material [45]. Another shared genus, *Lactococcus*, produces lactic acid during fermentation, enhancing nutrient absorption and potentially promoting gut health. Previously, Ma also found that Malayan pangolins contained bacteria capable of degrading chitin, as well as genera potentially involved in cellulose digestion, like *Cellulomonas*, *Lactococcus* and *Enterobacter*, all of which were also identified in our study. Overall, the functional roles and abundance patterns of bacterial genera in the gut microbiota of Chinese pangolins suggest a greater capacity for cellulose metabolism, whereas the gut microbiota of the Malayan pangolins appears to be more adapted to animal-based diets. This finding contradicted a previous hypothesis about the dietary preferences of Malayan pangolins. Previous research found that the gut microbiota of wild Malayan pangolins is rich in *Bacteroides* and *Clostridium*, and the gut microbiome of Malayan pangolins is similar to that of herbivores, capable of metabolizing carbohydrates and amino acids, reflecting the microbiota’s ability to ferment polysaccharides. We speculate that this difference may be due to the long-term absence of cellulose in their artificial diets during early captivity [46], highlighting the profound influence of environmental factors on gut microbiota composition and the lasting consequences of improper dietary management.

Despite the distinct natural diets and habitats of Chinese pangolins (native to southern China) and Malayan pangolins (native to southeast Asia) [47], their adaptation to captivity, including potential dietary adjustments, likely contributed to the observed differences in the gut microbiota composition. These compositional differences translated into distinct biological functions, as evidenced by disparities in the KEGG pathway and CAZyme profiles [48]. The Malayan pangolins displayed a higher relative abundance of pathways related to cellular processes and human disease, possibly due to their prolonged close contact with humans in captivity. In contrast, the Chinese pangolins exhibited more functions related to organismal systems and genetic information processing, suggesting an ongoing adaptation process to the captive environment. This study examined the functional traits of the gut microbiota using KEGG and CAZyme analyses. However, these results are largely based on predicted functions inferred from bacterial taxa. To gain a clearer understanding of the microbiota’s metabolic activities and their effects on host health, further validation using metabolomic or transcriptomic approaches would be beneficial.

The gut microbiota plays a pivotal role in regulating the host’s immune system, with its composition and functionality directly influencing the immune status, immune cell development, and pathogen defense. In this study, the higher abundance of potential pathogens in Malayan pangolins indicated a declined trend in immune function. The accumulation of antibiotic resistance and virulence genes in long-term captive animals poses significant challenges to their health and survival. Notably, our research uncovered significant differences in antibiotic resistance strategies between the gut microbiota of the two species, with Malayan pangolins exhibiting more efficient antibiotic resistance than Chinese pangolins. However, this study did not explore the specific genetic mechanisms underlying antibiotic resistance, nor did it assess potential sources of antibiotic exposure. Further research into the genetic basis of resistance and its association with the captive environment is crucial for developing effective disease management strategies.

However, this study had several limitations. First, the sample size was small, with only four pangolins per species, including just one female, which might have prevented a full assessment of the impact of sex differences on the results. Considering the potential sex-related differences in immunity, metabolism, and other factors, future research should increase the sample size and ensure gender balance to more comprehensively evaluate the impact of sex on the gut microbiota. Second, although we hypothesized that the captive environment and diet might influence the microbiota community, the lack of baseline data of wild pangolins prevented us from directly comparing the microbiota before and after captivity. We can only infer the causes of microbiota changes based on existing literature and observational results. To enhance the reliability of this study, future research should consider collecting fecal samples from wild pangolins to compare the microbiota community directly between the wild and captive animals. In general, because of the small sample size, variability in captivity durations, and differences in pre-captive conditions, this study should be treated as a preliminary case study. However, it provided foundational insights into the gut microbiota of pangolins and laid the groundwork for future research with larger and more representative samples. Looking ahead, further studies should consider how to better understand the impact of captivity on the pangolin gut microbiota and overall health with larger sample sizes and incorporating multidisciplinary approaches, such as immunological analysis and long-term monitoring. Additionally, exploring the relationship between microbiota changes and pangolin diseases or immune responses will help provide more scientific guidance for pangolin conservation and captive management.

Given the essential role of gut microbiota in host metabolism, nutrition, and immunity, alterations in its composition and function could have profound implications for pangolin health. Conservation efforts in the future should prioritize environmental enrichment and dietary improvements, potentially incorporating probiotics to modulate the gut microbiota, alleviate chronic stress, and mitigate the adverse effects of long-term captivity.

## 5. Conclusions

This study highlighted significant compositional and functional differences in the gut microbiota of captive Chinese and Malayan pangolins. The findings emphasize the potential impacts of captivity duration and diet on gut microbial diversity and functionality. Different pangolin species may exhibit different nutritional metabolism, functional composition, and immune responses due to differences in their gut microbiota, affecting their living conditions. These findings underscore the impact of captivity and diet on gut microbiota composition, although they must be interpreted cautiously given the small sample size, gender imbalance, and lack of pre-captive data. Despite these limitations, this study provides a foundation for future research, emphasizing the need for larger, longitudinal studies to further explore the effects of captivity on pangolin gut health.

## Figures and Tables

**Table 1:** Basic information and routine physiological parameters of eight captive pangolins.

	MP5	MP15	MP17	MP19	MJ13	MJ18	MJ25	MJ27
Gender	Male	Male	Male	Female	Male	Male	Male	Male
Species	*M. pentadactyla*	*M. pentadactyla*	*M. pentadactyla*	*M. pentadactyla*	*M. javanica*	*M. javanica*	*M. javanica*	*M. javanica*
Captivity duration (year)	2.5	2.5	2	2.5	5	5	5	5
Maturity	Adult	Adult	Adult	Adult	Adult	Adult	Adult	Adult
Body weight (kg)	6.68	7.46	9.16	5.6	9.96	9.14	6.54	6.35
Body length (cm)	47	53	55	36	65	64	57	53
Tail length (cm)	32	35	34	23	53	47	43	37
Rectal temperature (°C)	31.43 ± 1.54	31.58 ± 1.25	31.20 ± 1.09	32.01 ± 1.85	31.44 ± 1.51	31.89 ± 1.48	32.45 ± 1.78	30.68 ± 1.66
Heart rate (beats/min)	57.50 ± 7.48	59.50 ± 10.48	56.60 ± 2.22	57.90 ± 9.19	58.80 ± 5.89	55.30 ± 4.13	56.90 ± 6.24	58.70 ± 6.30
Respiratory rate (breaths/min)	21.70 ± 1.16	21.80 ± 1.14	22.10 ± 1.52	22.40 ± 1.78	21.00 ± 0.47	21.10 ± 0.89	21.90 ± 2.22	21.90 ± 1.55
Health status	Healthy	Healthy	Healthy	Healthy	Healthy	Healthy	Healthy	Healthy

**Table 2:** Alpha diversity indices of eight captive pangolins.

Samples	Chao1	Shannon	Simpson	ACE
MP5	7100.904	5.197	0.983	7038.848
MP15	6964.906	4.701	0.971	6898.871
MP17	7102.242	5.206	0.981	7054.912
MP19	6164.520	3.353	0.853	6104.062
MJ13	6026.771	3.167	0.805	5930.006
MJ18	6845.826	3.808	0.872	6733.518
MJ25	6909.893	2.479	0.636	6824.279
MJ27	3509.821	2.135	0.619	3451.216

## Data Availability

The original contributions presented in this study are included in this article. Further inquiries can be directed to the corresponding author(s).
